# Tumor-to-tumor metastasis from appendiceal adenocarcinoma to an ovarian mature teratoma, mimicking malignant transformation of a teratoma: a case report

**DOI:** 10.1186/s13000-019-0865-6

**Published:** 2019-08-14

**Authors:** Mitsutake Yano, Tomomi Katoh, Tetsuya Hamaguchi, Eito Kozawa, Mei Hamada, Koji Nagata, Masanori Yasuda

**Affiliations:** 1 Department of Pathology, Saitama Medical University International Medical Center, 1397-1 Yamane, Hidaka-City, Saitama, 350-1298 Japan; 2 Departments of Obstetrics and Gynecology, Oita University Faculty of Medicine, 1-1 Idaigaoka, Hasama-machi, Yufu-shi, Oita 879-5593 Japan; 3 Department of Gastroenterology, Saitama Medical University International Medical Center, 1397-1 Yamane, Hidaka-City, Saitama, 350-1298 Japan; 4 Department of Diagnostic Radiology, Saitama Medical University International Medical Center, Saitama, 350-1298 Japan

**Keywords:** Tumor-to-tumor metastasis, Appendiceal adenocarcinoma, Ovarian teratoma, Malignant transformation, Case report

## Abstract

**Background:**

Tumor-to-tumor metastasis (TTM) is a rare but well-documented phenomenon that is defined as metastasis in a histologically distinct tumor. Ovarian mature teratomas (OMTs) can coexist with various cancers by malignant transformation, which may make it difficult to distinguish these from TTM. Herein, we report a case of TTM from appendiceal adenocarcinoma to the OMT, mimicking the malignant transformation of OMT.

**Case presentation:**

A 67-year-old Japanese woman underwent abdominal total hysterectomy and bilateral salpingo-oophorectomy for an ovarian tumor in another hospital. She was initially diagnosed with mucinous carcinoma/carcinoid arising in the OMT. One year after surgery, she was referred to our hospital after the presentation of increased appendiceal mass. Cecal biopsy targeting an appendiceal tumor revealed scattered mucinous cells with signet ring features, which were morphologically similar to the malignant components in the previously diagnosed right OMT. Both the appendiceal adenocarcinoma and malignant components of the OMT stained positive for CK7, CK20, CDX-2, and SATB2 but negative for estrogen receptor, progesterone receptor, and pax-8. Finally, we confirmed the diagnosis of appendiceal goblet cell carcinoid metastasizing to the right OMT. The patient had tumor-bearing survival due to systemic chemotherapy administered for 35 months after the initial surgery.

**Conclusions:**

Awareness of the TTM phenomenon is important to avoid an incorrect diagnosis and to select the appropriate therapy when unusual malignancy is encountered in the OMTs.

## Background

Ovarian mature teratomas (OMTs) are common tumors that account for 20% of all ovarian neoplasms. An OMT is a germ cell tumor composed of cells derived from at least two, but frequently all three, germ cell layers. Although OMTs are usually benign, 1–3% undergo malignant transformation [1]. Adenocarcinoma arising in OMTs is the second most common malignancy after squamous cell carcinoma and accounts for 7% of all cases, with most tumors arising from the gastrointestinal and respiratory-type epithelium [2]. Metastatic adenocarcinoma from various organs is also often encountered in the ovary. Approximately 15% of all malignant neoplasms of the ovary are of the metastatic origin, and the gastrointestinal tract is the most common primary site (39%), followed by the breast (28%) and uterine endometrium (20%) [3]. The presence of an ovarian mass is sometimes the first manifestation of clinically occult non-ovarian primary cancer [3]. Therefore, when adenocarcinoma is encountered in OMTs, we need to decide whether it is primary or metastatic.

Tumor-to-tumor metastasis (TTM) is a rare but well-documented entity that is defined as a metastasis in a histologically distinct tumor. This phenomenon was first reported in 1902, and since then, approximately 200 cases have been reported [4]. The most frequent metastatic donors are lung carcinomas, followed by carcinomas of the breast, gastrointestinal tract, prostate, and thyroid, whereas the most frequent recipients are renal clear cell carcinomas, followed by sarcomas, meningiomas, and thyroid neoplasms [4–6]. TTM involving an ovarian tumor as the recipient is unusual, and only 13 cases have been described to date. OMTs, in particular, can coexist with various cancers via malignant transformation, making it difficult to distinguish these from TTM. Herein, we report a case with TTM, in which the OMT was a donor of appendiceal adenocarcinoma, mimicking the malignant transformation of OMT.

## Case presentation

**Fig. 1:**
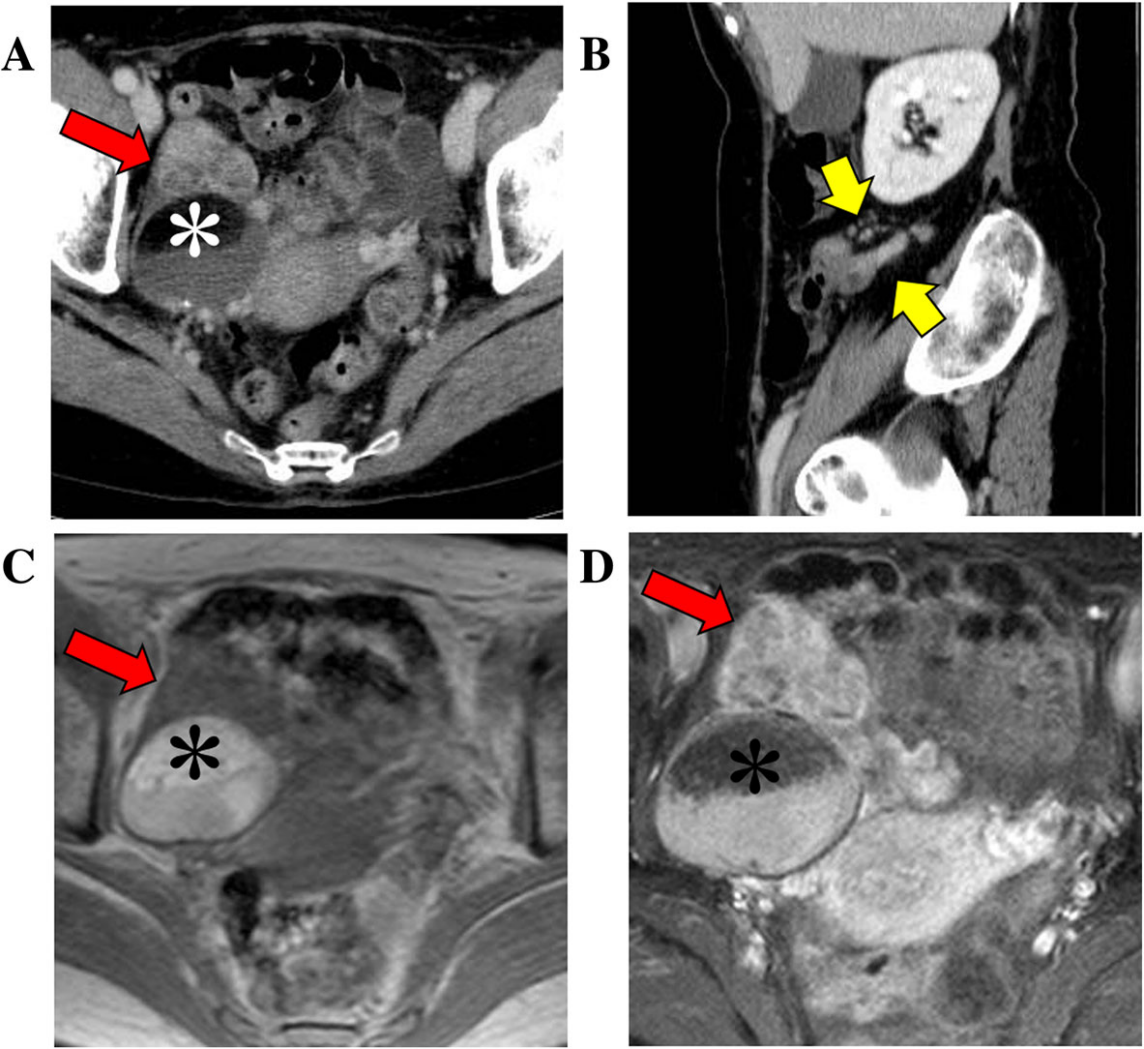
Clinical imaging. Enhanced computed tomography reveals the presence of: **a** a 70-mm right ovarian mass with a 40-mm round mass with fat (white asterisk) and solid component with non-uniform enhancement at the ventral side (red arrow) and (**b**) a 10-mm enlarged appendix with strong enhancement (yellow arrows). Magnetic resonance imaging of (**c**) T1-weighted image and (**d**) Fat-saturated T1-weighted image with gadolinium reveals fat (black asterisks), water, and solid components (red arrows)

A 67-year-old Japanese woman (gravida 2, para 2) with no past cancer history had visited another hospital with lower right abdominal pain. Enhanced computed tomography revealed the presence of a 70-mm mass with non-uniform enhancement at the right ovary and a 10-mm enlarged appendix with strong enhancement, for both of which OMT and appendicitis were suggested (Fig. 1a, b). No mass was detected in her breasts. Magnetic resonance imaging showed the ovarian mass had a fat, hair ball, and solid components and torsion of vessels, consistent with teratoma with malignant transformation (Fig. 1c, d). Serum levels of the tumor markers squamous cell carcinoma antigen, carbohydrate antigen 125, carbohydrate antigen 19–9, and carcinoembryonic antigen were 0.9 ng/mL, 9.5 U/mL, 12.8 U/mL, and 1.4 ng/mL, respectively. Cervical cytology was negative for intraepithelial lesion or malignancy. The patient underwent abdominal total hysterectomy with bilateral salpingo-oophorectomy. The intraoperative findings showed that the right ovarian tumor was twisted 720° and the appendiceal tumor was not clear. Her initial diagnosis was mucinous carcinoma/carcinoid arising in the OMT. A year after surgery, she was referred to our hospital because of an increased appendiceal mass. Cecal biopsy was performed. Finally, we were led to the diagnosis of appendiceal goblet cell carcinoid metastasizing to the right OMT, wherein the appendiceal cancer had already invaded the surrounding lymph nodes. Complete tumor resection was found impossible. The patient has had a tumor-bearing survival of 35 months after initial surgery due to systemic chemotherapy.

### Pathologic findings

**Fig. 2:**
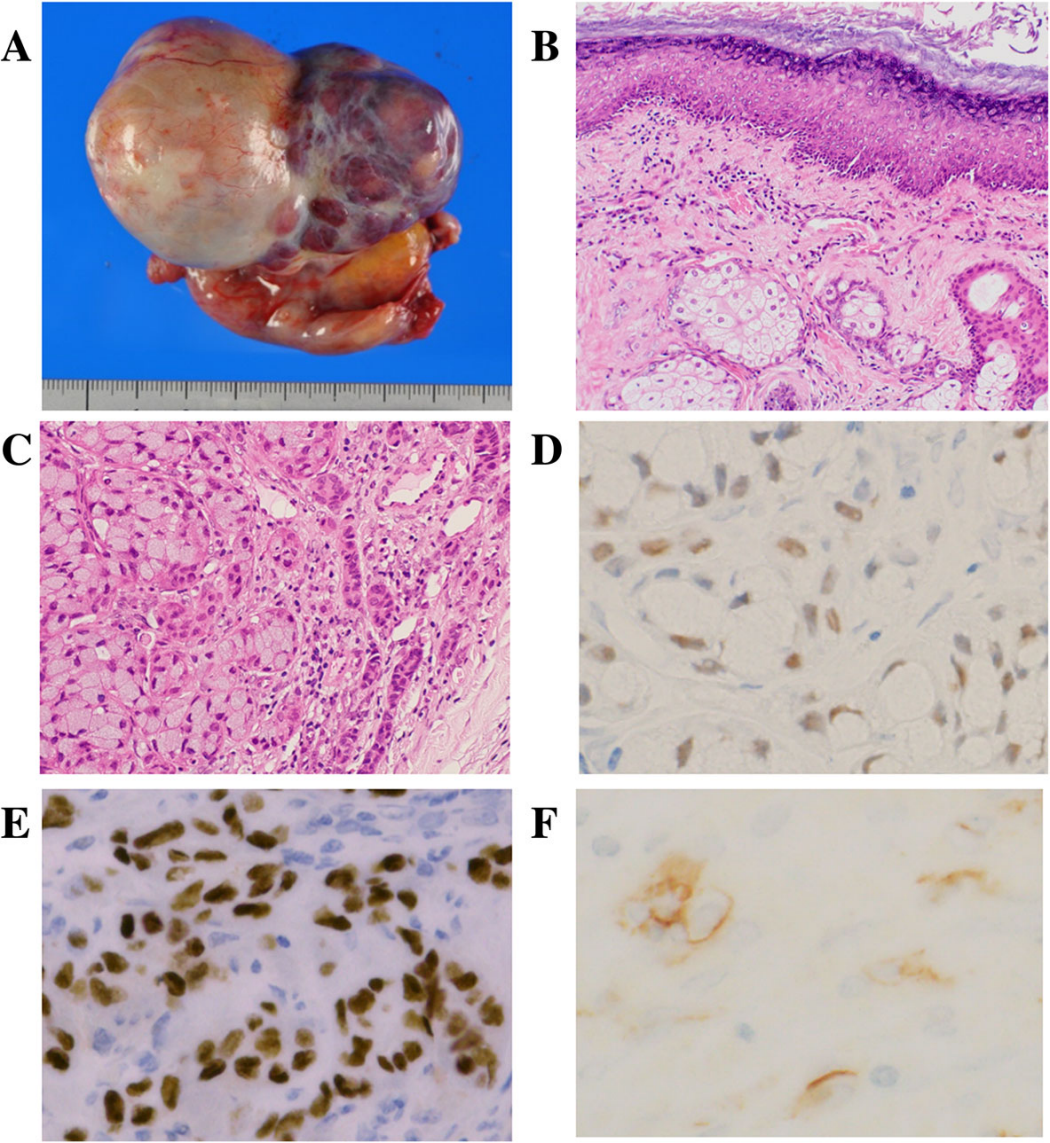
Right ovarian tumor. **a** Macroscopic examination shows a cystic tumor filled with greasy, yellow sebaceous material and a firm, well-demarcated, white nodule. **b** Microscopic examination shows the cystic wall covered with keratinizing stratified epithelium (H&E). **c** Microscopic examination shows the firm nodule consisted of neoplastic glandular components that contained scattered mucinous cells with signet ring features, focally forming small clusters without distinct glandular lumens (H&E). Immunohistochemical staining shows (**d**) CDX-2, (**e**) SATB2, and (**f**) CD56 (focal) positivity

The right oophorectomy specimen measured 55 × 35 × 35 mm, and the ovary was almost fully occupied by a cystic tumor filled with greasy, yellow, and sebaceous material and hair. There was a firm, well-demarcated, white nodule in the cyst (Fig. 2a, b). 7 blocks were examined for 55-mm ovarian mass. Microscopic examination showed that the inner surface of the cystic wall was lined by keratinizing stratified epithelium, with associated sebaceous glands and hair follicles resembling the normal skin accompanied by hyperemia (Fig. 2c). Mature adipose tissue was found throughout the cyst wall. These findings were consistent with those of an OMT. The OMT contained small amount of respiratory epithelium but not gastrointestinal epithelium. In addition, the firm nodule consisted of neoplastic glandular components that contained scattered mucinous cells with signet ring features, focally forming small clusters without distinct glandular lumens (Fig. 2d). These malignant components showed positive immunoreaction for CK7, CK20, CDX-2, SATB2 and CD56 (focal) but were negative for estrogen receptor, progesterone receptor, Pax-8, synaptophysin, and chromogranin A (Fig. 2e, f).

**Fig. 3:**
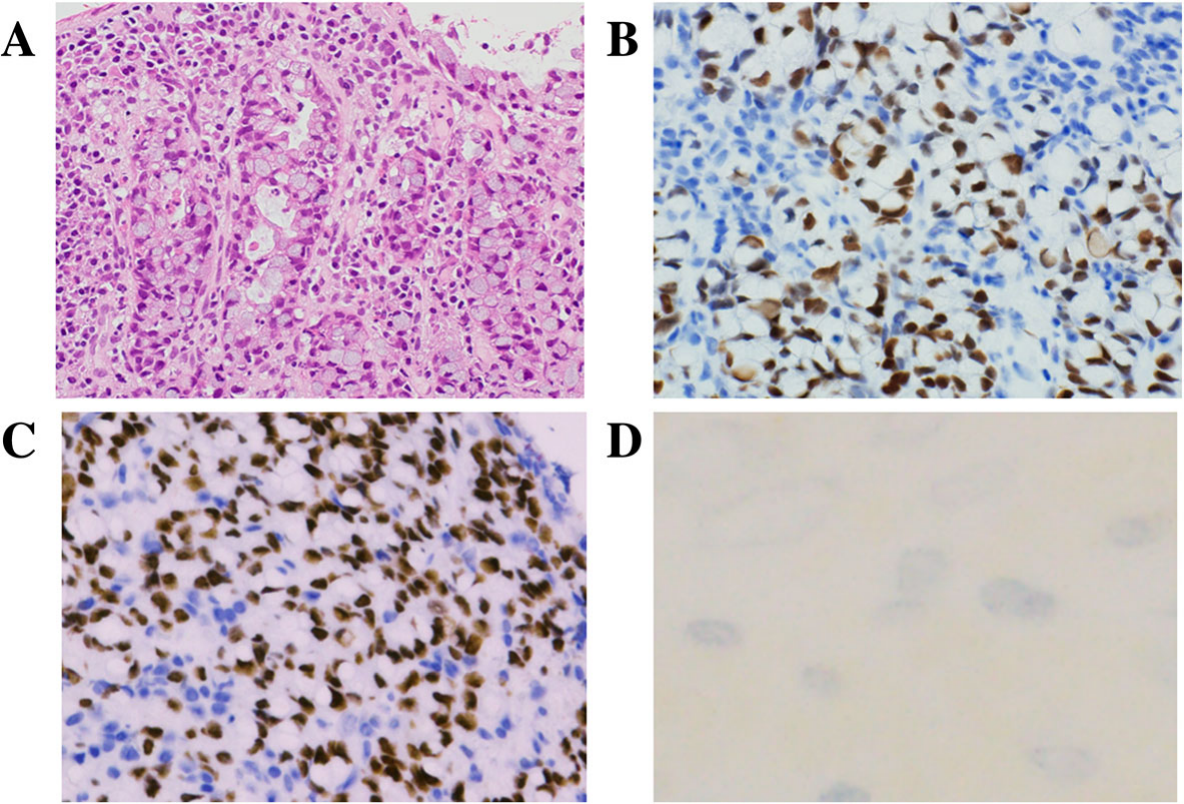
Appendiceal adenocarcinoma. **a** Microscopically, the cecum biopsy shows a neoplastic glandular proliferation (H&E). Immunohistochemical staining shows (**b**) CDX-2 and (**c**) SATB2 positivity, but (**d**) CD56 negativity

Cecum biopsy targeting the appendiceal tumor was performed because of a suspected OMT with malignant transformation or metastatic adenocarcinoma from the digestive system. Mucinous cells with signet ring features, similar to the malignant components previously observed in the right OMT, were also observed in the biopsy specimen (Fig. 3a). The appendiceal adenocarcinoma also showed positive immunoreaction for CK7, CK20, CDX-2, and SATB2 but was negative for estrogen receptor, progesterone receptor, Pax-8, CD56, synaptophysin, and chromogranin A (Fig. 3b). Based on these findings, the patient was diagnosed with appendiceal goblet cell carcinoid with metastasis to the right OMT.

## Discussion and conclusions

In the present case of a menopausal woman with an appendiceal mucinous/goblet cell carcinoid metastasizing to the right OMT, a somatic malignant transformation of the OMT was initially considered as a differential diagnosis. We diagnosed as appendiceal cancer to OMT metastasis from OMT with malignant transformation because the morphological similarity of both, the commonality of highly specific immunohistochemistry in the colon (CDX-2 and SATB2), and the absence of gastrointestinal epithelium in OMT. Mucinous carcinoid of the ovary histologically resembles mucinous/goblet cell carcinoid of the appendix. Baker et al. [7] reported that in 12 of 17 cases with primary ovarian mucinous/goblet cell carcinoids, an additional tumor characteristic of ovarian origin was seen. Of these, OMT was seen in seven cases, including mucinous carcinoma in three cases and mucinous borderline tumor and borderline Brenner tumor in one case each. Baker et al. [7] suggested that when an ovarian tumor contains a mucinous carcinoid with the neoplasm being characterized by an ovarian origin, primary mucinous carcinoid can be definitively diagnosed. However, in spite of our patient’s initial diagnosis of mucinous carcinoma/carcinoid as a primary tumor arising in the OMT, her primary tumor was detected later in the appendix during follow-up. In contrast to fewer than 30 cases of primary mucinous carcinoid of the ovary [7, 8], many reports of metastatic appendiceal goblet cell carcinoid of the ovary have been described in the literature [9]. We believe that histological confirmation of the appendix is necessary, even if a mucinous carcinoid contains extrinsic neoplasms of ovarian origin.

TTM is a well-documented phenomenon, for which a misdiagnosis of the primary organ may occur. TTM involving an ovarian tumor as the recipient is unusual, with only 13 cases described to date (Table 1) [4, 10–21]. Table 1 shows that the age of the 13 TTM patients ranged from 37 to 68 years, with an average of 53.6 years. The most frequent metastatic donors were breast carcinoma (7/14, 50%), followed by carcinoma of the cervix (3/14, 21%), gastrointestinal tract, and lung, whereas the most frequent recipients were OMTs (3/14, 21%) and granulosa cell tumors (3/14, 21%), most of which were benign (8/14, 57%) or borderline malignancies (4/14, 29%). Five of eight patients (63%) with available outcomes experienced a recurrence of disease or died. Based on these results, we believe that it is necessary to exclude TTM when malignancy of an ovarian tumor as a primary is in question. When OMT coexists with various cancers by malignant transformation, it is often difficult to distinguish TTM from OMT with malignant transformation. Therefore, a close clinical examination is recommended to rule out the breast and gastrointestinal tract as the origins of the ovarian metastasis. Van Rompuy et al. [8] showed that the immunohistochemical profiles in terms of CK7, CK20, CDX-2, and Pax-8 are considered to be useful in these differential diagnoses. When OMT shows a coexistence with squamous cell carcinoma or adenocarcinoma, cervical cytology examination is recommended above all. Positive findings in both immunohistochemical staining for p16^INK4a^ and in situ hybridization of human papillomavirus supports the diagnosis of a cervical cancer origin [11].

**Table 1:** Tumor-to-tumor metastasis to ovarian tumor

Case (year)	Age	From	Ovarian tumor			Prognosis
			Size (mm)	Histology	Side	
The present case (2019)	67	Appendiceal adenocarcinoma	55	Mature teratoma	Right	AWD
Zhang M (2018)	45	Cervical squamous cell carcinoma	81	Endometriotic cyst	Left	NA
Santos F (2018)	51	Cervical adenocarcinoma	110	Mature teratoma	Left	NED
Shi L (2015)	41	Gastric signet ring cell carcinoma	100	Granulosa cell tumor	Bilateral	DOD
Arnould L (2002)	63	Breast carcinoma	185	Granulosa cell tumor	Left	NED
Kirova YM (1999)	47	Breast carcinoma	80	Mature teratoma	Right	DOD
Perry LJ (1996)	68	Breast carcinoma	180	Fibroma	Right	NA
Twaalfhoven FCM (1994)	63	Breast carcinoma	220	Mucinous carcinoma	Bilateral	NA
Rasmussen RB (1992)	54	Cervical squamous cell carcinoma	54	Serous borderline tumor	Right	NED
Finn WG (1991)	68	Ileal carcinoid	NA	Adenocarcinoma	Left	NA
Rosa GD (1985)	40	Breast carcinoma	90	Thecoma	Right	NA
Mazur MT (1984)	53	Lung adenocarcinoma	NA	Granulosa cell tumor	Right	NA
Smale LE (1980)	37	Breast carcinoma	150	Benign Brenner tumor	Bilateral	DOD
Hines JR (1976)	53	Breast cystosarcoma phyllodes	150	Benign Brenner tumor	Right	DOD

Abbreviations: *NA* not available, *AWD* alive with disease, *NED* no evidence of disease, *DOD* death of disease

In conclusion, we report a case of TTM wherein an appendiceal adenocarcinoma metastasized to an OMT, mimicking the malignant transformation of OMT. To the best of our knowledge, the present case is the first case of TTM due to the metastasis of appendiceal adenocarcinoma to an OMT. Awareness of this phenomenon is important to avoid an incorrect diagnosis and to select the appropriate therapy when unusual malignancies in OMTs are encountered.

## Data Availability

All data generated or analyzed during this study are included in this published article.

## Abbreviations

OMT: Ovarian mature teratoma
TTM: Tumor-to-tumor metastasis
